# Regulation of endoplasmic reticulum functions

**DOI:** 10.18632/aging.100851

**Published:** 2015-11-18

**Authors:** Takushi Namba

**Affiliations:** Science Research Center, Kochi University, Kochi 783-8505, Japan

**Keywords:** endoplasmic reticulum, p53, IRE1α

The endoplasmic reticulum (ER) is a multifunctional organelle that is specialized for lipid synthesis, Ca2+ homeostasis, and protein synthesis. Protein synthesis is a multistep process involving folding and manipulation of proteins as well as the exportation of proteins to an appropriate location. Several stress responses, such as metabolic and anaerobic stress, induce ER dysfunction and protein unfolding, which accelerates the disruption of ER homeostasis. To prevent the production of unfolded proteins, cells induce the unfolded protein response (UPR), which is a highly regulated protein quality surveillance system. UPR maintains and restores ER homeostasis to increase the secretory function of the ER via the induction of the ER chaperone protein. This stimulates re-folding or degradation of unfolded proteins. However, irreparable ER stress induces a cell death response to eliminate these damaged cells [1]. Three types of ER transmembrane proteins, a protein-kinase and site-specific endoribonuclease, inositol-requiring enzyme 1 α (IRE1α), protein kinase R-like ER kinase/pancreatic eukaryotic initiation factor 2 kinase, and activating transcription factor 6 are essential for the mammalian UPR system. In particular, IRE1α is an essential signal transducer involved in the maintenance of ER function. The IRE1α pathway induces X-box binding protein 1(S) [XBP1(S)], which is the active form of XBP1 generated by IRE1α-dependent splicing of the XBP1 mRNA. XBP1(S) is a transcription factor that increases the expression of the ER chaperone, thus enhancing the secretory function of the ER and suppressing ER stress-mediated cell death [1].

Cancer cells alternate between glycolysis and protein synthesis pathways to survive and proliferate during stress associated with tumor growth and prolonged metabolic and anaerobic stress [2]. Cancer cells produce a lot of new proteins to satisfy their rapid growth using an energy source, such as the cancer-specific aerobic glycolysis pathway. Thus, the augmented function of the ER and resistance of ER stress-mediated cell death is essential for tumor proliferation and survival. Many reports have shown the enhancement of ER function in a variety of human cancers, but the mechanism for the cancer cell-specific regulation of ER function remains unresolved.

Our recent study reveals that the tumor suppression gene, p53, is a key regulator of ER function [3]. To the best of our knowledge, this is the first report to connect the cancer-related gene to ER function. p53 is mutated in at least half of human cancers, and defects in the p53 response lead to tumor development. The functions of p53 influence many types of cell signaling transduction pathways, but the role of p53 in the ER is largely unknown [4]. We found that the loss of p53 function increased IRE1α, XBP1(S), and ER chaperone expression in non-stressed and ER-stressed conditions and also increased ER function. Interestingly, IRE1α was abundantly expressed in mutant p53 cell lines compared with wild-type p53 cell lines. The upregulation of IRE1α expression triggered the activation of the IRE1α/XBP1 pathway. Further, our experiments identified the mechanism for increased IRE1 expression with p53 deficiency, and our results revealed that suppressed p53-dependent IRE1α-synoviolin-1 (SYVN1) association induced IRE1α proteasomal degradation. Therefore, wild-type p53 suppresses ER function and adapts to ER stress by keeping IRE1α expression levels low, which highlights the anti-cancer effects of p53. However, loss of p53 function activates the IRE1α/XBP1 pathway and stimulates resistance to ER stress-mediated cell death. Thus, up-regulation of IRE1α expression via the loss of p53 function is one of the mechanisms by which a cancer-specific increase in ER function and activation of the IRE1α/XBP1 pathway is related to some parts of the aggressive cancer phenotype found in p53-mutant cancers.

Another important finding of our current study is that an inhibitor of IRE1 selectively prevents the growth of p53-null tumors in vivo compared with that of wild type p53 tumors [3]. Several reports suggested that the inhibition of the IRE1α/XBP1 pathway is a new anti-cancer target. Cubillos-Ruiz et al. reported that the inhibition of activated XBP1 stimulates tumor-infiltrating dendritic cell-induced anti-tumor immunity [5]. The IRE1α inhibitor has an anti-cancer effect on human multiple myeloma and pancreatic cancers [6, 7]. Furthermore, DNA-damaging agents are widely used as treatments for various forms of cancer in the clinic, and its anti-cancer effects depend on the appropriate function of wild-type p53. Thus, mutant p53 cancer cells have been reported to show chemoresistance to many anti-cancer agents, and there is a need for novel therapies against mutant p53 cancers. Therefore, preventing the activation of the IRE1α/XBP1 pathway is a promising target for anti-tumor agents of mutant p53 cancers.

In summary, cancer cells need to increase ER function to survive stressful environments. Loss of p53 function augments ER function via the activation of the IRE1α/XBP1 pathway by the disruption of the p53-SYVN1-IRE1α complex. Thus, targeting the IRE1α/XBP1 pathway has potential as a novel therapeutic strategy for malignancy and chemo-resistance of mutant p53 cancers.
